# Novel reassortant of H9N2 avian influenza viruses isolated from chickens and quails in Egypt

**DOI:** 10.14202/vetworld.2021.2142-2149

**Published:** 2021-08-20

**Authors:** Moataz Elsayed, AbdelSatar Arafa, Shahira Abdelwahab, Amro Hashish, Ahmed Youssef

**Affiliations:** 1 Reference Laboratory for Veterinary Quality Control on Poultry Production, Animal Health Research Institute, P.O. Box 264, Dokki, Giza 12618, Egypt; 2 Department of Virology, Faculty of veterinary medicine, Suez Canal University, Ismailia, 41522, Egypt; 3 Department of Veterinary Diagnostic and Production Animal Medicine, College of Veterinary Medicine, Iowa State University, Ames, IA 50011, USA; 4 Animal Hygiene and Zoonoses, Faculty of Veterinary Medicine, Suez Canal University, Ismailia, 41522, Egypt

**Keywords:** genes, H9N2, influenza, mutations, poultry, reassortment

## Abstract

**Background and Aim:**

Poultry infections with H9N2 avian influenza viruses (AIVs) are endemic in Egypt. This study determined the genetic changes in the sequences of H9N2 AIVs isolated from chicken and quails in Egypt, including determining genetic reassortment and detecting the main genetic changes in hemagglutinin (HA) and neuraminidase (NA) genes.

**Materials and Methods:**

Swab samples were collected from chicken and quails, examined through reverse transcription-polymerase chain reaction, and AIVs from positive samples were isolated in embryonated chicken eggs. Complete genome sequencing and phylogenetic analyses were conducted for two H9N2 AIV isolates, and sequences of HA and NA gene segments were analyzed in another two isolates.

**Results:**

A novel reassortant virus was identified from a commercial chicken flock (A/chicken/Egypt/374V/2016) and quails from a live bird market (A/quail/Egypt/1253V/2016). The reassortant viruses acquired four genome segments from the classic Egyptian H9N2 viruses (HA, NA, NP, and M) and four segments from Eurasian AIVs (PB2, PB1, PA, and NS). Many genetic changes have been demonstrated in HA and NA genes. The isolated novel reassortant H9N2 virus from quails showed amino acid mutations in the antigenic sites on the globular head of the mature HA monomer matched with the parent Egyptian H9N2 virus.

**Conclusion:**

This work described the genetic characterization of a novel reassortment of the H9N2 virus in Egypt. The emergence of new reassorted AIV viruses and genome variability raises the concern of an influenza pandemic with zoonotic potentials.

## Introduction

Low pathogenic avian influenza virus (LPAIV) H9N2 was initially isolated from turkeys in the USA in 1966 [1]. Since then, LPAIV H9N2 was widely detected among domestic and wild birds worldwide [2]. Poultry infection with the LPAI H9N2 virus is prevalent in the Middle East and Asia [3,4]. Terrestrial poultry, especially chickens and quails, play a key role in expanding the host range for AIVs [3,5,6]. LPAIV H9N2 has been recorded as zoonoses and recognized as one of the potential pandemic threats [7,8].

H9N2 AIVs have been classified into two major genetic lineages: The North American and Eurasian lineages [9]. The Eurasian line was further divided into three sublineages represented by their prototype strains: A/chicken/Korea/38349 (Korean like), A/duck/Hong Kong/Y280/9 (Y280-like), and A/quail/Hong Kong/G1/97 (G1-like) [10]. From 1998 to 2010, the LPAIV H9N2 isolates from Central Asia and the Middle East were clustered into four distinct groups (A, B, C, and D) [11]. The emergence of new strains of influenza viruses might occur because of antigenic shift and drift [12]. Recently, many reassortment events increased AIVs that can cross the species barrier with humans, which might play a role in the emergence of novel zoonotic AIVs [13].

In Egypt, besides the circulation of the HPAI AIV H5 subtypes among poultry with occasional human infection since 2006, H9N2 AIV was first detected in quails in 2011 [14]. The cocirculation of AIV subtypes H9N2, H5N1, and H5N8 among poultry in Egypt was a severe challenge to the field of poultry production [15]. Genetic variability in the hemagglutinin (HA) gene of LPAIVs H9N2 isolated from the quails in Egypt, including antigenic sites, giving rise to the emerged variant cluster [16]. Moreover, a novel reassortant H9N2 virus emerged among pigeons in 2014 [17]. Furthermore, genetic changes in the neuraminidase (NA) gene of avian influenza H9N2 isolated from chicken and quails in Egypt have been reported [18]. Simultaneously, progressive evolution of the H9N2 subtype in the Middle East region has been indicated [18,19].

This study aimed to determine the genetic variability in H9N2 AIVs isolated from chickens and quails in Egypt and determine the main genetic changes in HA and NA genes.

## Materials and Methods

### Ethical approval

Ethical approval for this study was obtained from Animal Health Research Institute of Egypt.

### Study period and location

The study was conducted from January 2014 to December 2016. The collection of samples was conducted as part of an ongoing long-term AIV surveillance. The surveillance of H9N2 AIV covered four governorates (Behaira, Luxor, Monofiya, and Ismailia governorates). The samples were processed at the Laboratory of Virology (Reference Laboratory for Veterinary Quality Control on Poultry Production), Animal Health Research Institute, Giza, Egypt.

### Sampling

Cloacal and oropharyngeal swab samples were collected from apparently healthy domestic poultry from commercial chicken farms (n=144), chicken in the backyard (n=45), and quails in live bird markets (LBMs) (n=16) from four governorates of Egypt. The swabs were collected on 1 mL of sterile phosphate buffer saline and were transported quickly under freezing conditions to the Laboratory.

### Polymerase chain reaction (PCR) examination

The collected samples were centrifuged at 500× *g* for 10 min. Total RNA was extracted from the collected samples using a QiaAmp Viral RNA Mini Kit (QIAGEN, Hilden, Germany) according to the manufacturer’s instructions. Extracted RNA was subjected to reverse transcription PCR (RT-PCR) amplification using One-Step Real-Time RT-PCR Kit (QIAGEN, Germany) for detecting M, H9, H7, and H5 genes of influenza A and Newcastle disease virus (NDV) using specific sets of primers and probes [20-23].

### Virus isolation

An amount of 0.1 mL of the PCR-positive pooled samples to H9 subtype from each species (chicken and quails) was propagated in the allantoic fluids of 10-day-old specific-pathogen-free embryonated chicken eggs. The inoculated eggs were then incubated at 37°C for 24-48 h. After incubation, the inoculated eggs were chilled at 4°C for 3 h before harvesting the amnio-allantoic fluid. A second RT-PCR for detecting the M gene of the H9 subtype was conducted on the harvested allantoic fluid to confirm successful virus isolation as described previously [22]. The preparation of the RT-PCR mixture and the thermal conditions were conducted as described in the original research.

### Sequencing and phylogenetic analysis

PCR assays were used for the amplification of HA and NA segments of two isolates (A/chicken/Egypt/53RSF/2014 and A/chicken/Egypt/102VL/2015) as per the protocol of Adel *et al*. [16]. Nucleic acid sequencing of HA and NA genes was conducted for each amplified gene using Big Dye Terminator Kit (v.3.1; Applied Biosystems, Foster City, CA, USA) on a 3130 Genetic Analyzer (Applied Biosystems).

Of the examined samples, complete genome sequencing was conducted for two viruses isolated in 2016 (“A/chicken/Egypt/374V/2016 and A/quail/Egypt/1253V/2016”) using Ion Personal Genome Machine (PGM) sequencing system (Thermo Fisher Scientific, CA, USA). Reverse transcription and PCR amplification were conducted using the PathAmpTM FluA Reagents (Cat. No.4485019). Prepare template positive ion spheres particles containing clonally amplified DNA using Ion PGM™ Template OT2 200 Kit (Cat. No. 4480974) and the Ion OneTouch™ 2 instruments (Thermo Fisher Scientific). Sequencing was conducted with the Ion PGM™ Sequencing 200 Kit v2 (Cat. No. 4482006). The reagents used in sequencing were ordered from Life Technologies, Carlsbad, CA, USA, and used as per the manufacturer’s instructions.

Genetic analysis of sequences obtained from the selected viruses was matched with other reference strains retrieved from the GenBank database. The assembled sequences were analyzed using BLAST analysis of the National Center for Biotechnology Information. Multiple sequence alignment was conducted using BioEdit v.7.0 [24]. The pairwise nucleotide percent identity matrix for H9N2 isolates from Egypt and other countries was calculated using the CLUSTAL-V algorithm in the MegAlign program of the Lasergene software suite (DNASTAR, Madison, Wisconsin, USA). Sequence alignments of nucleotide and deduced amino acid sequences were conducted using the CLUSTAL-W program. Phylogenetic and molecular evolutionary analyses of each of the six gene segments of two complete genomes sequenced viruses and HA and NA genes of four viruses were conducted by the neighbor-joining method using MEGA v.8 [25]. The robustness of the groupings in the neighbor-joining analysis was assessed with 1000 bootstrap replicates. Three-dimensional structures of HA monomer of A/chicken/Egypt/374V/2016 and A/quail/Egypt/1253V/2016 were created by PyMOL v.1.1 (DeLano Scientific LLC, USA).

## Results

### Virus isolation and PCR detection

A total of four isolates were isolated in embryonated chicken eggs (two from chicken farms, one from backyard chicken, and one from quails). All the H9N2 AIV isolates obtained from domestic chicken and quails were found positive for M and H9 genes of AIV and negative for H5 and H7 subtypes of AIV and NDV through RT-PCR. Subsequent virus isolation was confirmed by qPCR targeting the M gene and H9 subtype. Four isolates were randomly selected for sequencing (two full genome sequencing and two isolates for HA and NA genes) for selecting isolates that were designated as following: A/chicken/Egypt/53RSF/2014 isolate from a backyard; A/chicken/Egypt/102VL/2015 and A/chicken/Egypt/374V/2016 from commercial chicken farms; and A/quail/Egypt/1253V/2016 isolate from LBM in Ismailia.

### Phylogenetic analysis of the full genome

#### Gene reassortment from H9N2 AIV parent Egyptian strain

Phylogenetic analysis of A/chicken/Egypt/374V/2016 and A/quail/Egypt/1253V/2016 isolates revealed that four genes (HA, NA, NP, and M) were clustered with classical H9N2 viruses in Egypt, which referred to as Group 1 with viruses from Israel and Saudi Arabia besides recent strains from Iran, Pakistan, and Bangladesh (supplementary data/figure can be available from the corresponding author).

#### Gene reassortment from H9N2 AIV wild strain

Phylogenetic analysis of A/chicken//Egypt/374V/2016 and A/quail/Egypt/1253V/2016 isolates revealed that the other four gene segments (PB2, PB1, PA, and NS) were clustered with the novel reassortant H9N2 viruses in Egypt from pigeons in 2014 (A/pigeon/Egypt/S10409A/2014) and (A/pigeon/Egypt/S10408B/2014) (supplementary data/figure can be available from the corresponding author).

Based on the reassortment event detected in PB2, PB1, PA, and NS genes of whole sequenced viruses, Egyptian H9N2 viruses could be divided into two groups; the first group contains classic viruses (Gr1 – classic), whereas the second group contains the reassortant viruses (Gr2 – reassortant) (supplementary data/figure can be available from the corresponding author).

**Figure-1:**
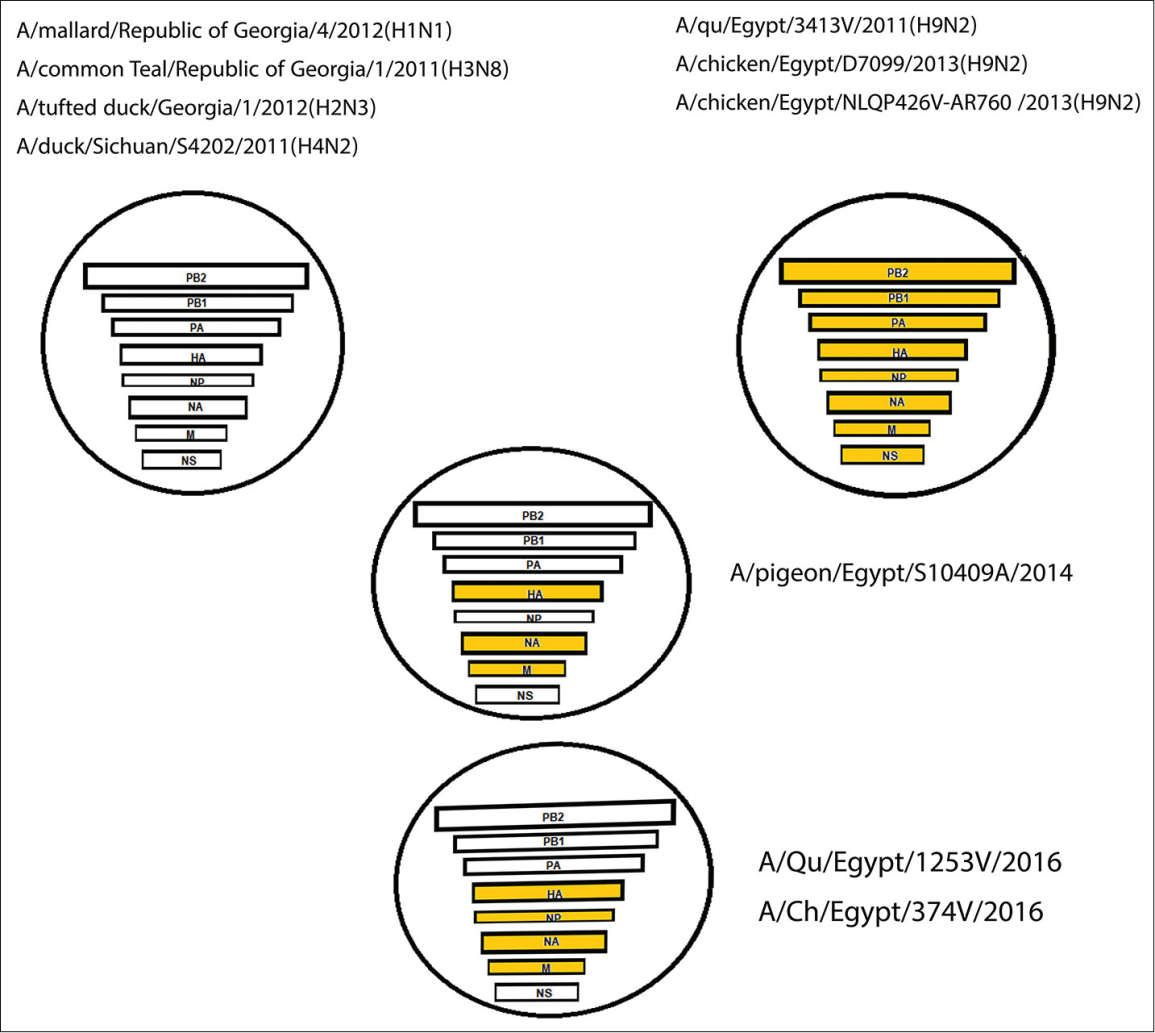
Schematic diagram of the novel reassortant Egyptian H9N2 virus.

The reassorted gene segments PB2, PB1, PA, and NS of the examined H9N2 viruses had the highest homology to the first reassortant H9N2 viruses isolated from pigeons in Egypt (A/pigeon/Egypt/S10409A/2014) and sub-branched from the Eurasian isolates from Georgia A/mallard/Republic of Georgia/4/2012 (H1N1), A/common teal/Republic of Georgia/1/2011 (H3N3), A/tufted duck/Georgia/1/2012 (A/H2N3), and A/duck/Sichuan/S4202/2011 (A/H4N2) (Figure-1).

### The percentage of nucleotide identity of the internal genes of two isolates with the ancestor viruses

The H9N2 AIV isolate originated from quail showed similarity to A/pigeon/Egypt/S10409A/2014 by a percentage of 98% in PB2 and 99% in PB1, PA, and NS genes. The chicken isolate was closely related to r by a percentage of 99% for the four genes (PB2, PB1, PA, and NS). However, a lower similarity percentage was recorded in the two isolates than A/quail/Egypt/3413V/2011, which was related to the original group of Egypt H9N2 viruses. NP segments of the examined isolates were related to the parent Egyptian H9N2 viruses (nucleotide similarity ranged from 94% to 99%) and were not related to the reassorted NP segments of the pigeon isolates (nucleotide similarity ranged from 89% to 90%) (Table-1).

**Table-1:** Similarity percent among internal genes of parent viruses with the novel reassortant Egyptian H9N2 virus.

	A/quail/Egypt/1253V/2016	A/chicken/Egypt/374V/2016
PB2		
A/pigeon/Egypt/S10409A/2014	98%	99%
A/common Teal/Republic of Georgia/1/2011 (H3N8)	97%	98%
A/mallard/ Republic of Georgia/13/2011 (H6N2)	97%	98%
A/quail/Egypt/3413V/2011 (Group 1)	85%	85%
PB1		
A/pigeon/Egypt/S10409A/2014	99%	99%
A/mallard/Republic of Georgia/ 4/2012 (H1N1)	98%	98%
A/duck/Guizhou/1078/2011(H11N9)	97%	98%
A/quail/Egypt/3413V/2011 (Group 1)	83%	84%
PA		
A/pigeon/Egypt/S10409A/2014	99%	99%
A/wild waterfowl/Dongting/C2383/2012(H1N2)	98%	98%
A/greater white-fronted goose/Netherlands/1/2012 (H1N1)	98%	98%
A/quail/Egypt/3413V/2011 (Group 1)	88%	88%
NS		
A/pigeon/Egypt/S10409A/2014	99%	99%
A/tufted duck/Republic of Georgia/1/2012 (H2N3)	98%	99%
A/mallard duck/ Netherlands/18/2010 (H6N8)	98%	98%
A/quail/Egypt/3413V/2011 (Group 1)	87%	87%
NP		
A/pigeon/Egypt/S10409A/2014	90%	90%
A/pigeon/Egypt/S10408B/2014	90%	89%
A/chicken/Egypt/D7108E/2013 (Group 1)	99%	98%
A/quail/Egypt/113413V/2011 (Group 1)	94%	94%

### Genetic analysis of HA gene

**Figure-2:**
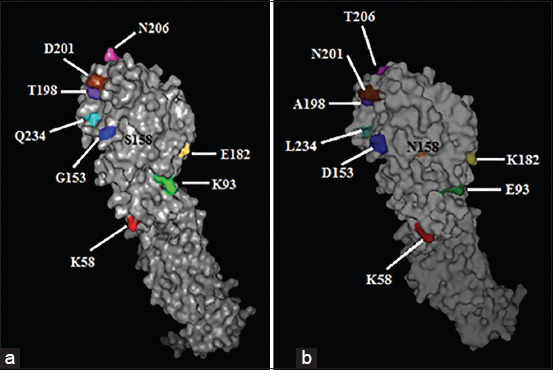
Three-dimensional structures of HA monomer of Egyptian H9N2 avian influenza virus created by PyMOL 1.1 program (DeLano Scientific LLC), the 3D molecular structures of 2 Egyptian isolates show the differences in the antigenic sites on the globular head of the mature HA monomer, (a) the molecular structure of HA protein of A/quail/Egypt/1253V/2016 and (b) the molecular structure HA protein of A/chicken/Egypt/374V/2016. Each amino acid residue labeled with different color.

Amino acid mutations at the antigenic epitopes revealed many critical transformations in the HA gene epitope sites with high variability in the quail isolate. The isolate from quails (A/qu/Egypt/1253V/2016) showed a mutation in the HA gene glycosylation site 206 to N-glycosylation sites and in the left edge of receptor binding site that located in amino acid residues at position 232-237 from NGLIGR to NGQIGR and in the binding RBS site at amino acid residues at position 110, 161, 163, 191, 198, 202, and 203 from PWTHALY to PWTH**T**LY (Table-2). In addition, the three-dimensional structure of the HA monomer of the reassorted quail isolate differed from the reassorted chicken isolate in the antigenic sites on the globular head of the mature HA monomer (Figure-2).

**Table-2:** Comparison of amino acid sequences of HA protein of H9N2 viruses in Egypt.

Strain	Cleavage Site	Glycosylation Site											RBS		
	
		29	105	141	145	196	206	207	218	298	305	492	Left edge^2^	Binding site^1^	Right edge^3^
A/qu/Egypt/113413V/2011	RSSRGLF	NST	NGT	NVT	NO	NO	NO	NO	NO	NST	NIS	NGT	NGLIGR	PWTHALY	GTSKS
A/ch/Egypt/53RSF/2014	RSSRGLF	NST	NGT	NVT	NO	NO	NO	NO	NO	NST	NIS	NGT	NGLIGR	PWTHALY	GTSKS
A/ch/Egypt/102VL/2015	RSSRGLF	NST	NGT	NVT	NO	NO	NO	NO	NO	NST	NIS	NGT	NGLIGR	PWTHALY	GTSKS
A/ch/Egypt/374V/2016	RSSRGLF	NST	NGT	NVT	NO	NO	NO	NO	NO	NST	NIS	NGT	NGLIGR	PWTHALY	GTSKS
A/qu/Egypt/1253V/2016	RSSRGLF	NST	NGT	NVT	NO	NO	**NDT**	NO	NO	NST	NIS	NGT	NG**Q**IGR	PWTH**T**LY	GTSKS
A/qu/Egypt/141267V/2014	RSSRGLF	NST	NGT	NVT	NGT	NO	NO	NTT	NO	NST	NIS	NGT	NG**QA**GR	PWTH**V**LY	GTSK**A**
A/qu/Egypt/D10106/2014	RSSRGLF	NST	NGT	NVT	NO	NTT	NO	NO	NO	NST	NIS	NGT	N**D**L**T**GR	PWTH**T**LY	GTSKS
A/ch/Egypt/F7297B/2013	**K**SSRGLF	NST	NGT	NVT	NO	NO	NO	NO	NO	NST	NIS	NGT	NGLIGR	PWTH**V**LY	G**K**SKS
A/qu/ Hk/G1/97	RSSRGLF	NST	NGT	NVT	NO	NO	**NDT**	NO	NRT	NST	NIS	NGT	N**D**L**Q**GR	PWTH**E**LY	G**I**S**RA**
A/du/Hk/Y280/97	RSSRGLF	NST	-	NV**S**	NO	NO	NO	NO	NRT	N**T**T	N**V**S	NGT	NGLQGR	PWT**NT**LY	GTSK**A**
A/du/Hk/Y439/97	**A**S**N**RGLF	NST	-	NVT	NO	NO	NO	NO	NRT	N**T**T	N**V**S	NGT	N**DQQ**GR	PWTH**E**LY	GTS**RA**
A/Hong Kong /1074/1999	RSSRGLF	NST	NGT	NVT	NO	NO	**NDT**	NO	NRT	NST	NIS	NGT	NGL**Q**GR	PWTH**E**LY	GTS**RA**
A/Guangdong/MZ058/2016	RSSRGLF	NST	NO	NV**S**	NO	NO	NO	NO	NO	N**T**T	N**V**S	NGT	NGL**M**GR	PWT**NT**LY	GTS**TA**

Amino acid residues in HA proteins of Egyptian H9N2 viruses compared to other avian and human H9N2 viruses expressed by H9-Numbering

1 Amino acid residues at positions 110, 161, 163, 191, 198, 202, and 203, respectively, ^2^Amino acid residues at position

2 32–237,

3 Amino acid residues at position 146–150

Changes indicated by bold

In comparison with the first isolate of the H9N2 virus from quails in 2011 (A/quail/Egypt/113413V/2011), the quail isolate in this study had nine amino acid mutations comparable with those in the antigenic epitopes A, B, C, D, and E (D153G and N158S at the antigenic site A; K182E, A198T, N201D, and T206N at the antigenic site B; M58K at the antigenic site C; L234Q at antigenic site D; and E93K at antigenic site E). In addition, three of these amino acids were located at the antigenic site II (D153G, N201D, and L234Q) and one at the overlapping site (T206N) (Table-3) [26,27,28].

**Table-3:** Amino acid mutations in HA antigenic epitopes of Egyptian H9N2 viruses.

	143^aΙ^	165^bΙ^	170^bΙ^	153^aΙΙ^	201^bΙΙ^	234^dΙΙ^	14^a^^	197^b^^	206^b^^	58^c^	93^e^	158^a^	182^b^	198^b^
A/qu/Egypt/113413V/2011	T	K	P	D	N	L	N	T	T	M	E	N	K	A
A/ch/Egypt/53RS/2014	-	-	-	-	-	-	-	-	-	K	-	-	-
A/ch/Egypt/102VL/2015	-	-	-	-	-	-	-	-	-	K	-	-	-	-
A/ch/Egypt/374V/2016	-	-	-	-	-	-	-	-	-	K	-	-	-	-
A/qu/Egypt/1253V/2016	-	-	-	G	D	Q	-	-	N	K	K	S	E	T
A/qu/Egypt/122313V/2012	-	-	-	E	G	-	-	-	I	-	-	-	-	V
A/qu/Egypt/141267V/2014	-	-	-	G	D	Q	-	-	-	-	-	-	-	V
A/qu/Egypt/14864V/2014	-	-	-	G	D	Q	-	-	-	-	-	-	-	V
A/qu/Egypt/D10106/2014	-	-	-	N	A	-	-	-	-	-	-	S	-	T
A/qu/Egypt/D10093/2014	-	-	-	N	A	-	-	-	-	-	-	S	-	T
A/pigeon/Egypt/S10409A/2014	-	-	-	-	-	-	-	-	-	-	-	-	-	-
A/ch/Egypt/15255VD/2015	-	-	-	-	-	-	-	-	-	K	-	-	-	-
A/ch/Egypt/D9377/2014	-	-	-	-	-	-	-	-	-	-	-	-	-	-
A/qu/Hk/G1/97	-	-	-	G	-	-	-	-	N	-	-	S	-	E

^Ι, ΙΙ, ^^The H9 antigenic epitopes according to Kaverin *et al*. [26]

^a, b, c, d, e^Amino acids related to antigenic epitopes group A, B, C, D & E of HA according to [27,28].

### Genetic analysis of NA gene

Interestingly, a new glycosylation site (NVT) at position 306 has been detected in A/quail/Egypt/1253V/2016 isolate that was not found in human H9N2 viruses. Glycosylation site 69 was NGT in all Egyptian viruses except in A/quail/Egypt/1253V/2016 was NST. No mutations were detected in the binding pocket residues involved in zanamivir and oseltamivir antiviral drug resistance. Moreover, no stalk deletion was detected in the NA segment. Besides, amino acid mutations were not detected in the drug-binding site of the NA gene segment (Table-4).

**Table-4:** Amino acid residues in neuraminidase NA protein.

Virus	Neuraminidase Active Site (HB)		Framework Site		Glycosylation Sites							
		
	366-373	399-406	431-433	425	44	61	69	86	146	200	234	306
A/quail/Egypt/113413V/2011	IKKDSRAG	DSDSWSGY	PQE	E	NTS	NIT	NGT	NWS	NGT	NAT	NGT	NO
A/chicken/Egypt/53RSF/2014	IKKDSRAG	DSDSWSGY	PQE	E	NTS	NIT	NGT	NWS	NGT	NAT	NGT	NO
A/chicken/Egypt/102VL/2015	IKKDSRAG	DSD**G**WSGY	PQE	E	NTS	NIT	NGT	NWS	NGT	NAT	NGT	NO
A/chicken/Egypt/374V/2016	IKKDSRAG	DSDSWSGY	PQE	E	NTS	NIT	NGT	NWS	NGT	NAT	NGT	NO
A/quail/Egypt/ 1253V/2016	IKKDSRAG	DSDSWSGY	PQE	E	NTS	NIT	N**S**T	NWS	NGT	NAT	NGT	**NVT**
A/duck/Egypt/C9787/2014	IKKDSRAG	DSD**G**WSGY	P**H**E	E	NTS	NIT	NGT	NWS	NGT	NAT	NGT	NO
A/chicken/Egypt/D5490B/2012	IK**E**DSRAG	DSD**G**WSGY	PQE	E	NTS	NIT	NGT	NWS	NGT	NAT	NGT	NO
A/quail/Hong Kong/G1/97	IKKDSR**S**G	DSD**IR**SG**S**	PQE	E	NO	NIT	N**N**T	NWS	NGT	NAT	NGT	NO
A/duck/HongKong/Y280/97	IK**E**DSR**S**G	DSD**N**WSGY	PQE	E	NO	NO	N**S**T	NWS	NGT	NAT	NGT	NO
A/duck/HongKong/Y439/97	I**S**KDSR**S**G	D**NNN**WSGY	PQE	E	NO	NIT	N**N**T	NWS	NGT	NAT	NGT	**NMT**
A/Hong Kong/1074/1999 (Human H9N2)	IKKDSR**S**G	DSD**N**WSGY	PQE	E	NO	NIT	N**N**T	NWS	NGT	NAT	NGT	NO
A/Hunan/44557/2015 (Human H9N2)	IK**NG**SR**S**G	**E**SD**D**WSGY	PQE	E	N**S**S	**Deletion**	N**S**T	NWS	NGT	NAT	NGT	NO

Amino acid residues in NA proteins of Egyptian H9N2 viruses compared to other avian and human H9N2 viruses expressed by H9-Numbering, Changes indicated by bold

## Discussion

Since the emergence of H9N2 AIV in quails in Egypt was first recorded in 2011 [14], the possibility of the genetic reassortment event of H9N2 genes has been proposed [19]. Two antigenic variants of H9N2 viruses have been detected among quails in Egypt. Those variant viruses were evolved from quails as a sporadic case in 2012 and further detection was reported in 2014 and 2015 [16,17]. This novel reassortant virus might have been introduced into domestic poultry in Egypt through the reassortant H9N2 viruses isolated from pigeons in 2014 “A/pigeon/Egypt/S10409A/2014 and A/pigeon/Egypt/S10408B/2014” [17].

In this study, phylogenetic analysis of sequences of the full genome of two H9N2 AIVs from chickens and quails and the sequences of HA and NA genes of other two viruses isolated from chicken revealed a novel reassortant H9N2 virus in chickens and quail. The reassortant viruses detected in this study showed that four genes were driven from the parent virus (HA, NA, NP, and M), and the other four segments (PB2, PB1, PA, and NS) were driven from the Eurasian strains. In the study of Kandeil *et al*. [17], the reassortant virus detected in pigeons had driven three genes (HA, NA, and M) from the classical H9N2 AIV that was seen in 2012, whereas the other five reassortant genes (PB2, PB1, PA, NP, and NS) had driven from the Eurasian AIVs that were detected later in Egypt. Therefore, the novel reassortant viruses detected in this study might originate from the Egyptian strains (G1) that were first seen in 2012 and may be generated from reassortment events between other subtypes of influenza in Egypt with the parent Egyptian H9N2 viruses. There is another possibility the reassortant H9N2 AIVs detected in this study partially shared the gene segments with the previously detected reassortant viruses in pigeons in 2014. Unlike the previously identified reassortant H9N2 AIVs in Egypt in pigeon in 2014, this study identified a novel reassortment event in the NP gene segment of the two strains of this study was like that found in the parent Egyptian H9N2 viruses.

Avian influenza H9N2 virus is circulating in Egyptian chicken farms [29]. The detection of the reassortant viruses in the commercial sector of poultry production represented by the isolate A/chicken/374V/2016 from a chicken farm from Monofya Governorate and A/quail/1253V/2016 from quail in an LBM in Ismailia Governorate indicated the spread of the reassortant viruses in different geographical areas in Egypt. The reassortment events of H9N2 AIVs in Egypt could be related to many factors such as high poultry population density and high prevalence of the disease, the absence of proper biosecurity measures, and close contact of domestic poultry with migratory birds [30]. In addition, the emerging of reassortant events might result in novel gene constellations [26].

The genetic evolution trend of H9N2 AIV is under the immune pressure of the vaccine [31]. In comparison with the first H9N2 virus isolated in 2011 (A/quail/Egypt/113413V/2011), many critical amino acid mutations at the antigenic epitope sites of the HA gene were identified. The three-dimensional structure of the HA monomer of the reassorted quail isolate (A/quail/Egypt 1253V/2016) differed from the reassorted chicken isolate (A/chicken/Egypt/374V/2016) in the antigenic sites on the globular head of the mature HA monomer. The quail isolate in this study had nine amino acid mutations comparable with those in the antigenic epitopes A, B, C, D, and E, according to Huang and Yang [32]. Moreover, the mutations in the HA antigen-binding sites that were detected in this study were not found in the reassortant H9N2 AIV isolate from a pigeon (A/pigeon/Egypt/S10409A/2014) and were variable in the other Egyptian strains. Three of these amino acids were located at the antigenic site II (D153G, N201D, and L234Q) and one at the overlapping site (T206N) [26,31-33]. These amino acid substitutions may affect viral interactions with cell surface receptors [34]. Moreover, it has been reported that such substitutions were frequent in escape mutants of the H9 subtype virus. Taken together, this indicated that the reassortant H9N2 viruses were more likely to have antigenic variability in HA antigenic sites. Mutations in the HA gene of H9N2 AIV might decrease the efficacy of H9N2 vaccine strains and increase the possibility of an emerging pandemic strain, including zoonotic strains [31].

The NA gene glycosylation sites were similar in most H9N2 viruses with seven glycosylation sites [1]. This study demonstrated much genetic variability in the NA gene in the quail isolate, detecting variability in two glycosylation sites in positions 69 and 306. This result was in line with the study of Mosaad *et al*. [18], who detected six glycosylation sites in the NA gene of the H9N2 subtypes isolated from Egypt (A/Q/Egypt/14864V/2014). The substitution mutations in the NA protein may affect the virus’s pathogenicity to poultry and transmissibility to humans.

Genetic variability was detected in amino acid levels in the surface genes indicated continuous evolution of H9N2 AIVs among poultry in Egypt. Therefore, regular large-scale surveillance of the H9N2 virus is important for monitoring the virus’s genetic changes among poultry in Egypt.

## Conclusion

This work identified continuous H9N2 virus evolution in Egypt with complicated genetic reassortment with other viruses and highlighted the importance of the continued implementation of H9 surveillance programs.

## Data Availability

Supplementary data can be available from the corresponding author on a reasonable request.

## Authors’ Contributions

AA, SA, and AY: Designed the study. ME and AY: Collected the samples and performed the laboratory work. ME, AY, and AH: Collected the data, performed data analyses and prepared the manuscript. AY: Revised the manuscript. All authors discussed the results and commented on the manuscript and approved the final version of the manuscript.
